# A novel vertebral trench technique (VTT) involving transforaminal endoscopic lumbar foraminotomy (TELF) for very highly up-migrated lumbar disc herniation above L5

**DOI:** 10.1186/s12891-021-04548-3

**Published:** 2021-08-14

**Authors:** Yong Yu, Ye Jiang, Fuling Xu, Yuhang Mao, Lutao Yuan, Chen Li

**Affiliations:** 1 Department of Neurosurgery, Zhongshan Hospital, Fudan University, Shanghai, 200032 China; 2 Department of Neurosurgery, Minhang Hospital, Fudan University, Shanghai, 201199 China

**Keywords:** Transforaminal endoscopic lumbar foraminotomy, Very highly up-migrated, Vertebral trench technique

## Abstract

**Background:**

Percutaneous endoscopic lumbar discectomy (PELD) has become popular for the treatment of symptomatic lumbar disc herniation (LDH). The very highly up-migrated lumbar disc herniation (VHUM-LDH) is difficult to remove under PELD. The purpose of this research is to investigate the feasibility, clinical efficacy and operative nuances of a novel VTT involving TELF for this type of herniation.

**Methods:**

Eleven patients with very highly up-migrated LDH who underwent VTT involving TELF discectomy from May 2016 to May 2019 were included in this study. The operative time, length of hospital stay, and postoperative complications were recorded. Pre-and postoperative radiologic findings were investigated. All the patients were followed more than 1 year. The visual analogue score (VAS), Oswestry Disability Index (ODI), Japanese Orthopaedic Association (JOA) scores and modified MacNab criteria were used to assess surgical efficacy.

**Results:**

All the 11 patients underwent successful surgery. We compared the VAS, ODI, and JOA scores before and after surgery. The differences were statistically significant (*P* < 0.05). According to the modified MacNab criteria, 10 patients were assessed as “excellent”, 1 patient was assessed as “good” at the last follow up.

**Conclusion:**

The novel VTT involving TELF discectomy is a supplement to the traditional PELD. This technique enables the endoscope with working cannula to get closer the sequestrated nucleus pulposus without irritating the exiting nerve root, and facilitates the nucleus pulposus be removed successfully under direct visualization. The VTT involving TELF discectomy can be a safe, effective and feasible surgical procedure for the treatment of LDH with very highly up-migrated.

## Background

Over the half past century, the field of spinal surgery has advanced significantly. In general, the trend is to minimize approach-related tissue trauma and, hence, reduce postoperative pain and disability [1]. Percutaneous endoscopic spine surgery corresponds to the trend. The obvious advantages of working-channel endoscopic spinal surgery are the reduction of the surgical corridor to less than 10 mm, avoiding soft tissue stripping and muscular denervation, minimizing bony resection to prevent iatrogenic instability and excellent visualization are part of that compromise [2, 3].

Since 1970s, the pioneers in percutaneous endoscopic spinal surgery have developed various techniques for the treatment of symptomatic lumbar disc herniation (LDH)through hard work [4–8]. The percutaneous endoscopic lumbar discectomy (PELD) has greatly improved over the past 10 years and gradually become popular for the treatment of LDH [9–13]. Although microscopic lumbar discectomy is considered as a gold standard for LDH, more and more studies have demonstrated PELD provides equivalent outcomes to microsurgical with better visualization, less soft tissue injury and shorter rehabilitation time [14–17]. The traditional PELD technique has two basic approaches: the transforaminal and interlaminar approaches. In the percutaneous transforaminal endoscopic lumbar discectomy (TELD) [16, 18], neural decompression is performed through intervertebral foramen, using the safe triangle of Kambin’s which lies between the exiting and the traversing root. At L5–S1 level, percutaneous interlaminar endoscopic lumbar discectomy (IELD) is preferred due to anatomical constrains like high iliac crest [19].

**Fig. 1:**
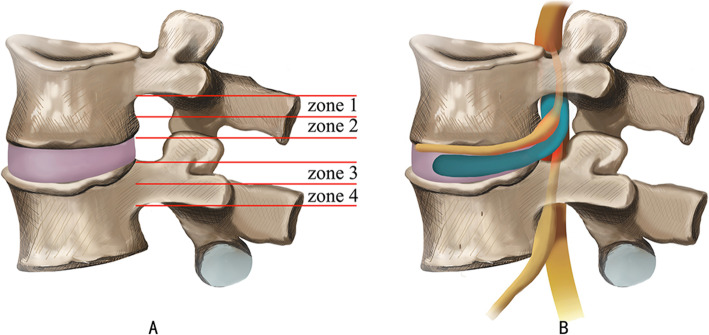
(**A**) Four anatomic zones and levels of disc herniation, as divided by Lee et al.: zone 1, the far-upward zone, within 3 mm of the lower edge of the upper pedicle. (**B**) in this article, the very highly up-migrated disc herniation represents that the migration of the herniated disc to the cephalad side beyond the level of the lower edge of the cephalad lumbar pedicle

With the development of endoscopic armamentarium and technological innovations, as well as a better understanding of endoscopic anatomy and approach, the range of indications for PELD is ever expanding [9, 10, 12]. However, some types of LDH are still considered to be challenging to achieve successful neural decompression under PELD, such as high-grade upward or downward migrated types [20]. Lee et al. divided the sagittal plane of the lumbar spine into 4 regions (Fig. 1A), zone 1 (within 3 mm of the lower edge of the upper pedicle) represents the far-upward zone and zone 4 represents the far-downward zone [21]. He reported that patients with high-grade migrated discs have less favorable outcomes than those with near migrated or non-migrated discs treated with conventional transforaminal PELD. However, due to the numerous advantages of PELD, spinal surgeons have never stopped exploring this technique. There are several modified approaches have been reported for high-grade migrated discs: transpedicular approach [22–24], posterior modified translaminar approach [25–27] and contralateral intervertebral foramen approach [28], etc. most of which for down-ward migrated discs.

Some authors further defined herniated discs above zone 1 as very highly up-migrated [27] (Fig. 1B). Obviously, the further the sequestrated nucleus pulposus (SNPs) upward migrate, the more difficult it be removed. The main reason is that the very highly up-migrated SNPs are hidden from endoscopic view by anatomic barriers like facets, inferior pedicles, and exiting nerve root. To the best of our knowledge, there are few relevant studies on TELD techniques for this type of herniation; furthermore, the clinical outcome is also not ideal. In this article, we want to present a novel vertebral trench technique (VTT) involving TELF. From Mary 2016 to May 2019, we used this technique to treat 11 patients with very highly up-migrated LDH. The technical points and short-term effects are summarized below. The purpose of this report is to validate the feasibility of the technique and describe several operative nuances and pearls based on our experiences.

## Methods

### Patient population

Between May 2016 and May 2019, 11 patients with very highly up-migrated disc herniation at L4-L5 or above L4–5 in our department were enrolled into this retrospective, clinical study. A novel vertebral trench technique involving transforaminal endoscopic lumbar foraminotomy was performed under local anesthesia. All operations were performed by the same senior author (Dr. Yu), who has specialized training in spinal neurosurgery and many years of experience in PELD.

Among the 11 patients, 6 were males, and 5 were females, with a mean age of 68.5 ± 10 years (range 58–78 years). The herniated disc was located at L2/3 in 1 patient, L3/4 in 4 patients, L4/5 in 6 patients (Table 1). The inclusion criteria were as follows: single-level lumbar disc herniation at L4-L5 or above L4–5, with very highly up-migrated disc herniation, verified by magnetic resonance imaging and computed tomography that the migration of the herniated disc to the cephalad side beyond the level of the lower edge of the cephalad lumbar pedicle; clinical symptoms and signs in accordance with imaging changes; unilateral radiating leg pain with or without positive Lasegue sign; and failure of standard conservative treatment for at least 3 months.

**Table 1:** Patients’ demographic characteristics and Operative characteristics

SEX		Age(years)	Treatment level			Operative time (mean) (range)	Hospital stay (mean) (range)
Male	Female	(Mean ± SD)	L2/3	L3/4	L4/5	54.90 ± 11.00 min (45–70 min)	2.55 days (2–3 days)
6	5	68.5 ± 10	1	4	6		

Exclusion criteria were: 1) lumbar spinal canal stenosis; 2) lumbar spondylolisthesis, or segmental instability suggested by radiographic findings; 3) vertebral infection, tumor or other vertebral lesions; 4) a surgical history involving the same level. 5) an inability to tolerate surgery due to other severe concurrent diseases.

### Ethics statement

This study was conducted in accordance with the guidelines of the 1964 Helsinki declaration and was approved by the ethics committee of Minhang Hospital, Fudan University. All of patients signed informed consent forms for surgery procedure.

### Endoscopic instruments

The endoscopic surgical system TESSYS (Joimax GmbH, Karlsruhe, Germany) was applied to perform the surgery, including an endoscope, endoscopic sheaths, 3-mm high-speed grinding drills, nucleus pulposus clamp, laminectomy rongeurs, etc. The radiofrequency probe (Trigger-Flex® Bipolar System, Elliquence LLC, Baldwin, NY, USA). was utilized to control bleeding and ablate.

### Clinical evaluation

Operative time, hospitalization time, and complications were recorded. Visual analog scale score for leg pain and back pain (range, 0–10), Japanese Orthopaedic Association Scores(range 0–29), and Oswestry disability index score (range, 0–100) were evaluated preoperatively and at the second day, 3, 6, and 12 months postoperatively. MacNab criteria were used to evaluate surgical effectiveness.

### Statistical analysis

SPSS 23.0 was used to study data for statistical analysis. For continuous variables that conform to the normal distribution, the VAS score, JOA score, and ODI are expressed as mean ± standard deviation (x ± s); The VAS score, JOA score and ODI at different time points were compared using repeated measures analysis of variance. *P* < 0. 05 is statistically significant.

### Surgical procedure

Surgical procedures were based on the conventional transforaminal endoscopic approach using transforaminal endoscopic surgical system (TESSYS [Joimax GmbH, Karlsruhe, Germany]) with full-endoscopic visualized drill technique [29].

**Fig. 2:**
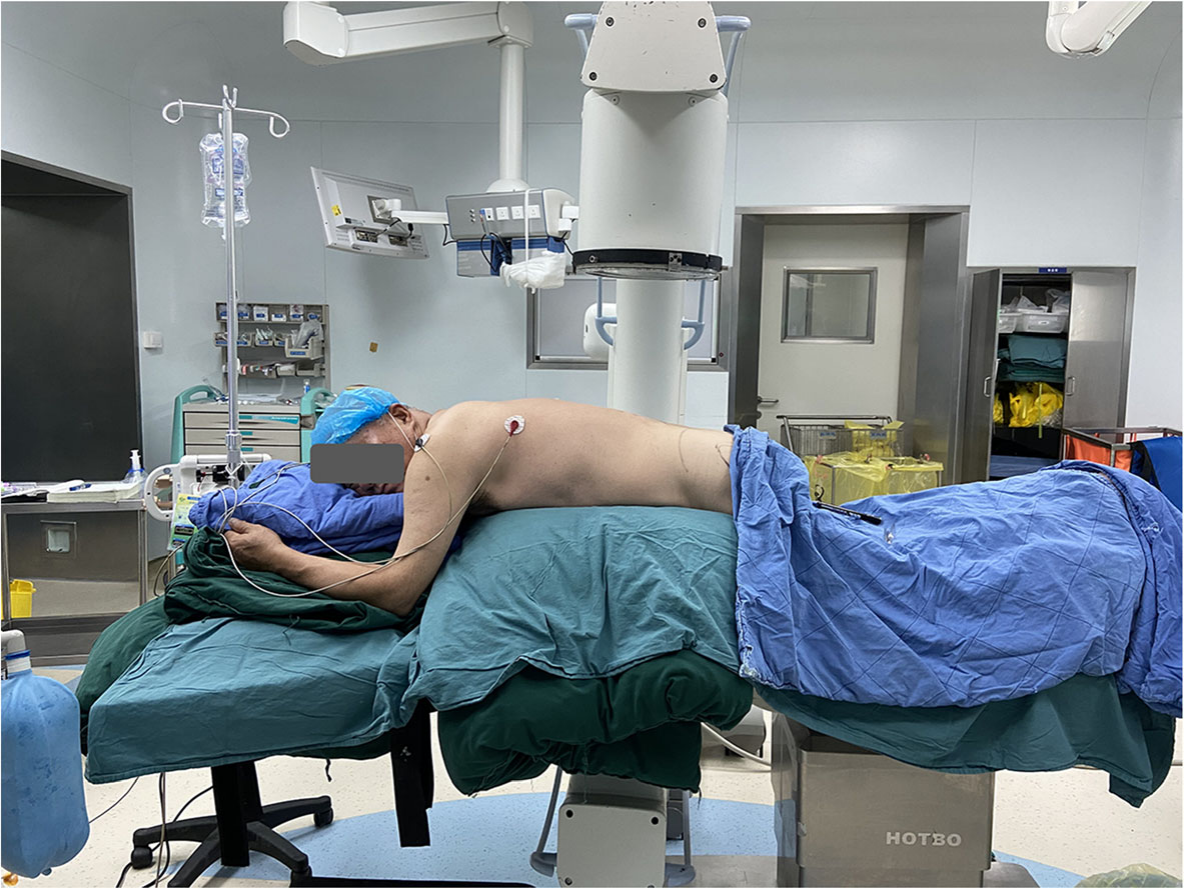
The patients were positioned on a radiolucent arch frame in the prone position with hips and knees in flexion and hands naturally oriented at the sides of the head

The patients were positioned on a radiolucent arch frame in the prone position with hips and knees in flexion (Fig. 2), which can decrease the local pressure of the intervertebral foramen [30]. The surgical procedures were performed under local anesthesia (1% lidocaine 10 mL,0.5% lidocaine and 0.125% ropivacaine 15–20 mL).

**Fig. 3:**
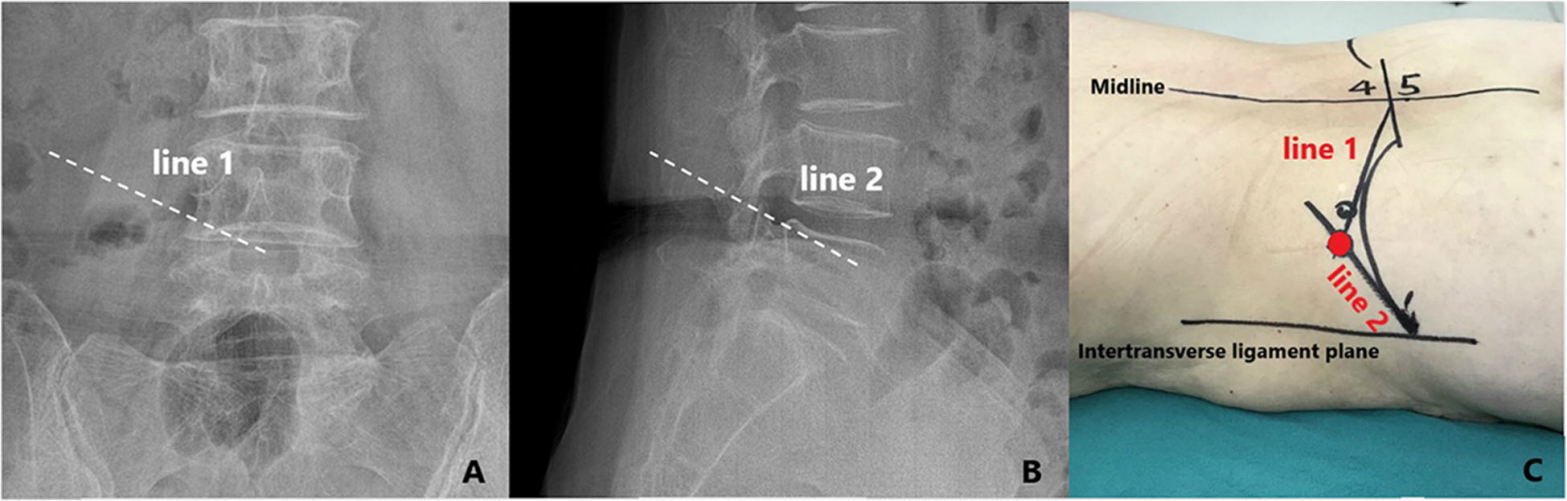
(**A**, **B**, **C**)The skin entry point was determined by the intersection of imaginary line 1 and line 2. line 1 was drawn on the AP Ferguson view that crosses the transverse disc plane from the center of the upper endplate of the inferior vertebra to the tip of superior articular process(SAP). on the lateral C-arm view, line 2 was drawn on the lateral skin that projects at the lower third of the foramen (posterior edge of superior endplate of caudal vertebra to the tip of superior articular process)

The skin entry point was determined by the intersection of imaginary line 1 and line 2 [11]. Using a metal rod as a radiopaque landmark, line 1 was drawn on the AP Ferguson view that crossed the transverse disc plane from the center of the upper endplate of the inferior vertebra to the tip of superior articular process(SAP). On the lateral C-arm view, line 2 was drawn on the lateral skin that projects at the lower third of the foramen (posterior edge of superior endplate of caudal vertebra to the tip of superior articular process) (Fig. 3) [11].

**Fig. 4:**
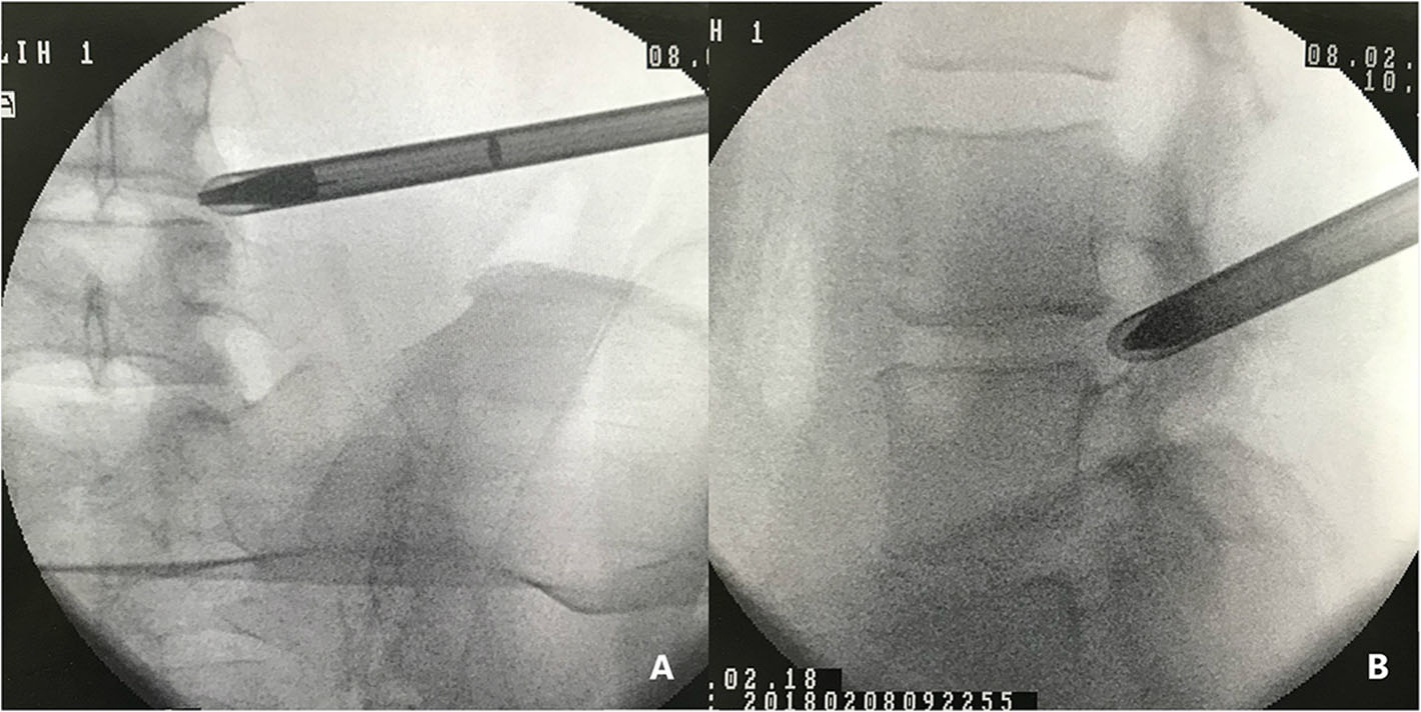
(**A**, **B**) The position of working cannula was controlled radiologically in two planes before the full-endoscopic visualized foraminotomy was performed

The patient was sterilized and draped routinely. After local anesthesia was achieved around the skin entry point, an 18-gauge spinal needle was introduced under fluoroscopic guidance, and the subcutaneous tissue, muscle layers and facet joint along the trajectory were infiltrated with 1% lidocaine solution about 10 mL. The ideal final position of the needle tip was at the lower third of the vertebra foramen. After infiltrating 15–20 mL of 0.5% lidocaine and 0.125% ropivacaine in the intervertebral foramen, Then the spinal needle was replaced with a guidewire. An 8 mm stab wound was made in the skin, a pencil-like puncture rod was inserted toward the intervertebral foramina over the guide wire, and the beveled working cannula was introduced into the lower third portion of the foramen along the pencil-like rod, with the opening facing toward the exiting nerve. The position of working cannula was controlled radiologically in two planes before the full-endoscopic visualized foraminotomy (Fig. 4). Finally, the endoscopic surgical system was introduced and then all the subsequent steps were performed under constant irrigation with excellent endoscopic visualization.

**Fig. 5:**
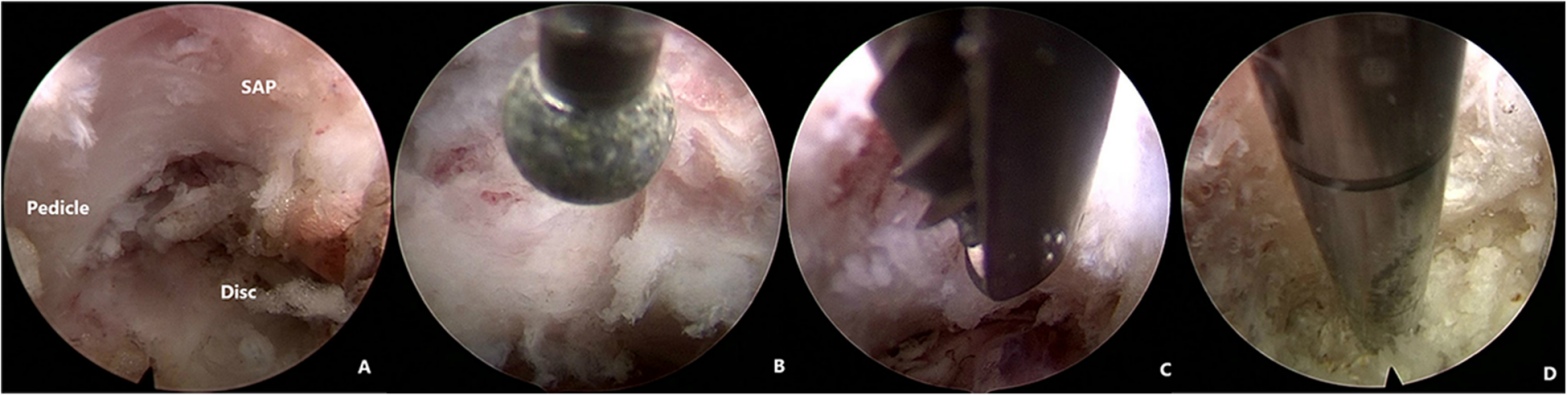
Full-endoscopic visualized foraminotomy. (**A**) The anatomical landmarks, such as the pedicle, superior articular process (SAP) of the inferior vertebra and disc were identified under excellent endoscopic visualization. (**B**, **C**, **D**) different kinds of instruments can be used to resect the ventral part of SAP of the inferior vertebra to enlarge the foramina. (**B**) Diamond drill; (**C**) The guarded endoscopic drill; (**D**) Ultrasound Bone knife

The anatomical landmarks, such as the pedicle, SAP of the inferior vertebra and disc were identified by palpation through the flexible curved tip of the radiofrequency probe. The ventral part of SAP of the inferior vertebra was partially resected by direction-variable drill or ultrasonic bone knife (Fig. 5 A, B, C, D). We performed the full-endoscopic visualized foraminotomy with the aid of excellent endoscopic visualization rather than with the aid of intervention technique. After foraminotomy, enough manipulation space is obtained. The endoscope can be advanced into the spinal canal and gradually turned to the cranial direction.

**Fig. 6:**
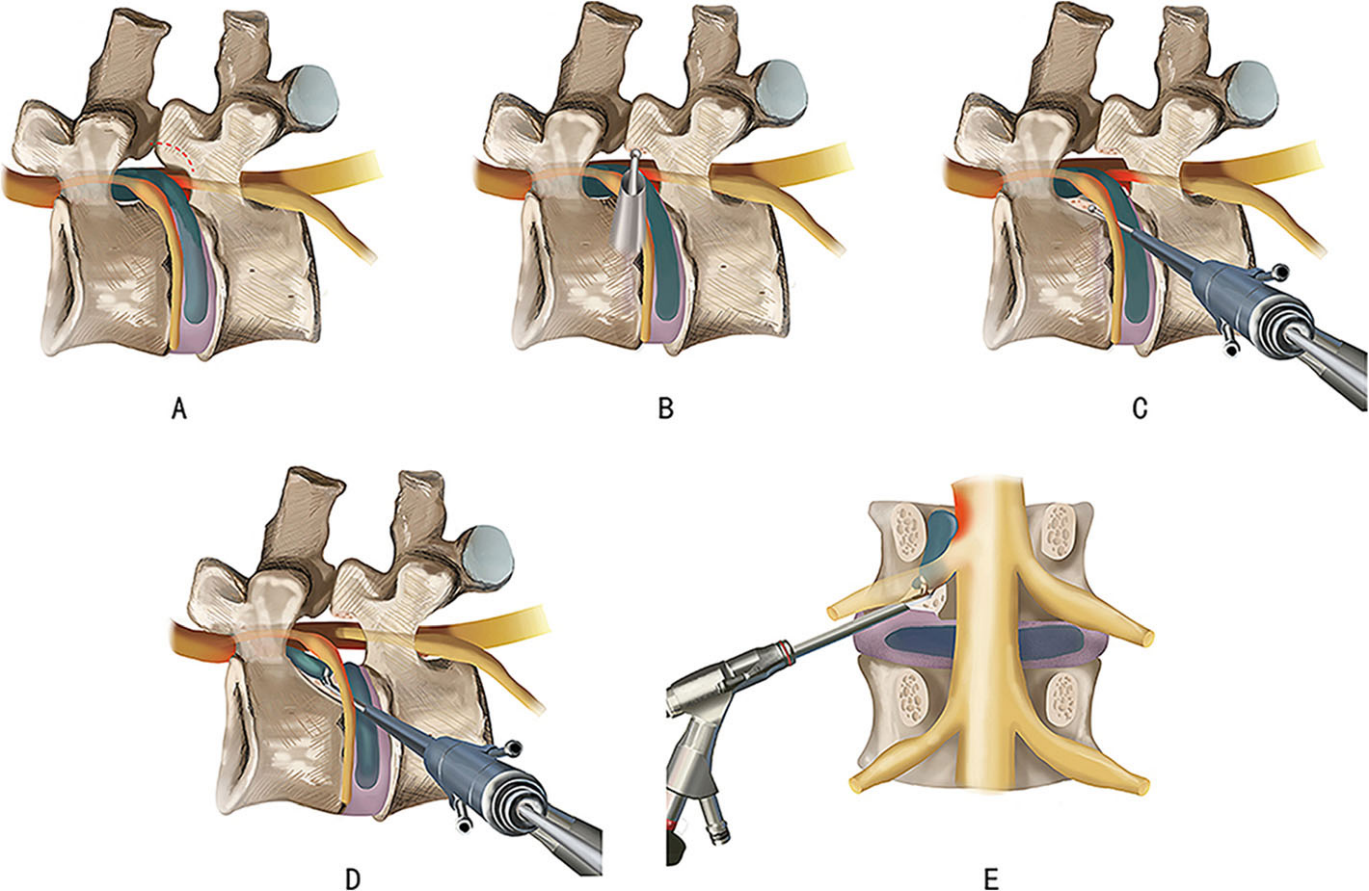
Illustrations show the VTT involving TELE to treat the very highly up-migrated lumbar disc herniation above L5 (**A**) The ventral part of SAP of the inferior vertebra which will be partially resected. (**B**) Full-endoscopic visualized foraminotomy was performed with the endoscopic diamond bur. (**C**) In the posterior surface of the cephalad vertebral above the inferior endplate, a diamond bur was used to dig a trench to the cranial direction. (**D**) and (**E**) the endoscope with working cannula moved into the trench. The sequestrated nucleus pulposus was removed with endoscopic forceps under direct vision

The endoscope with cannula was gradually shifted upwards with twisting motion till the exiting root was partially visible. Then it was rotated to the posterior surface of the superior vertebral body to follow the trail of the upward migrated and SNPs, which lied in the axilla and ventral of the exiting root usually. In the posterior surface of superior vertebral above the inferior endplate, diamond bur or small endoscopic trephine was used to dig a trench, which about 5 mm depth, to the cranial direction. Subsequently, the endoscope with working cannula moved into the trench. In this step, more space could be obtained to reduce the irritation of the exiting and traversing nerve root because of the trench (Fig. 6). The SNPs was explored and released carefully with a flexible bipolar radiofrequency probe which prevented nerve damage or tearing of the dura sac when removing it. It was manually dragged and removed with angled endoscopic forceps under direct vision (Fig. 6). After completely removing the herniated disc, exploration was repeated in different directions to confirm that no residual disc fragment was present in the spinal canal. The bipolar RF device was used to control the bleeding and ablate the rupture of the annulus of the intervertebral disc to seal the rupture site, which reduced the risk of recurrence. Normal pulsation of the nerve root with the Valsalva maneuver was a sign to end the surgery.

The postoperative specimen was routinely sent for pathological examination. The patient’s intraoperative condition, surgical tolerance, operative time and postoperative complications were recorded. Lumbar magnetic resonance imaging (MRI) was performed immediately after surgery to determine whether a residual nucleus pulposus remained and the effect of nerve root decompression.

### Postoperative management

The patients were allowed to ambulate with waist protection after eight hours bed rest and were instructed in rehabilitation training. Symptomatic treatment, including elimination of swelling and pain, was routinely applied postoperatively. Three weeks after surgery, the patients were allowed to engage in normal activities. A regular follow-up was performed.

## Results

### Demographic data

The patient’s demographic characteristics and operative characteristics were summarized in Table 1. The average operative time was 54.90 ± 11.00 min (range 45–70 min). The average hospital stay was 2.55 days (range 2–3 days). Among the 11 patients, no complications, such as nerve injury, CSF leakage or wound infection, occurred.

### Clinical outcomes

**Fig. 7:**
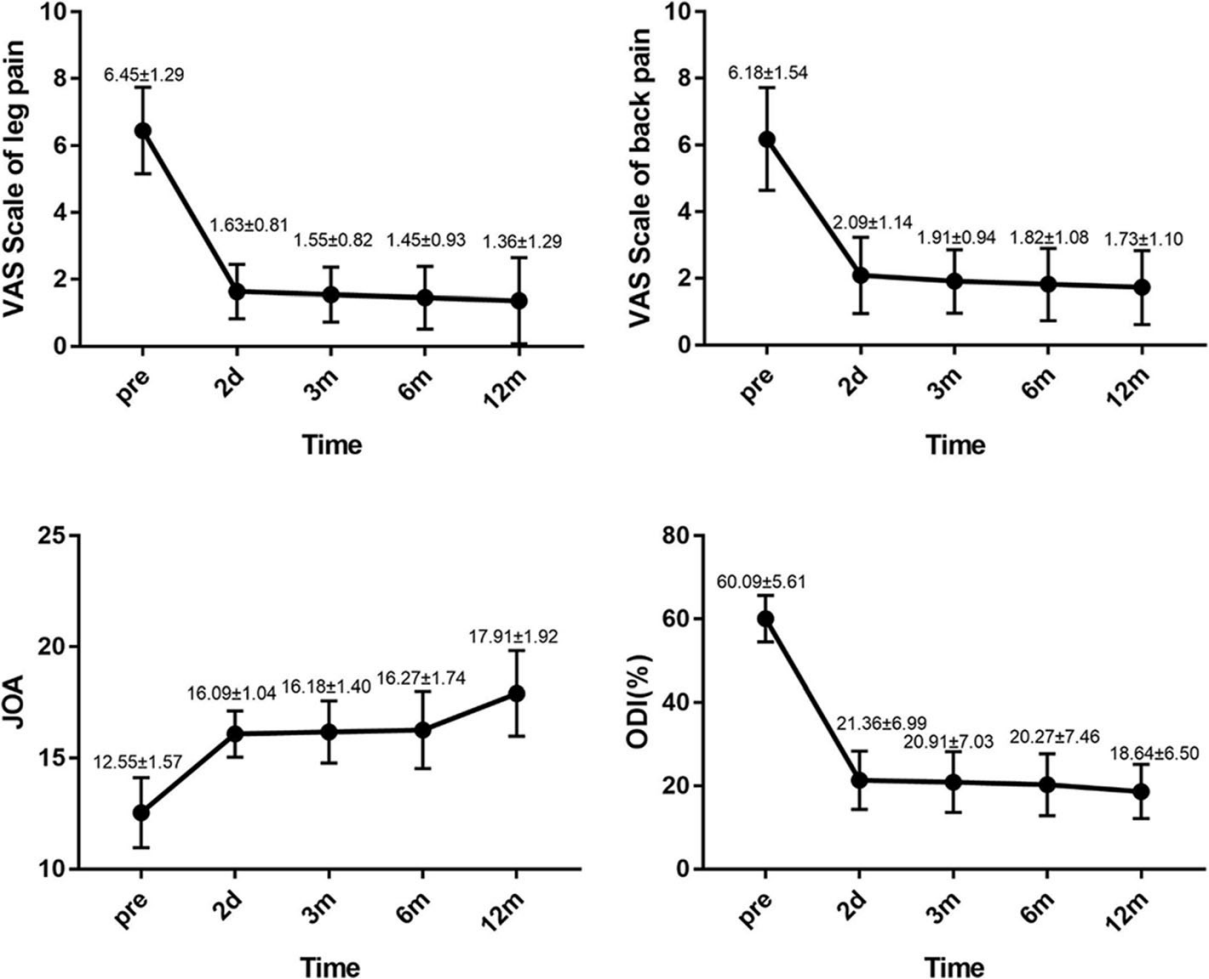
The mean values of visual analog scale (VAS) scores for leg and back pain, JOA scores, and Oswestry disability index (ODI) scores. Pre = preoperative; postop, 2d = 2 days after operation, 3 m = 3 months after operation, 6 m = 6 months after operation, 12 m = 12 months after operation

All the 11 patients underwent successful surgery and completed the follow-up visits for up to 12 months. The VAS leg pain was 6.18 ± 1.54 before surgery, 2.09 ± 1.14 at the second day, 1.91 ± 0.94 at 3 months, 1.82 ± 1.08 at 6 months and 1.73 ± 1.10 at 12 months after surgery. The VAS back pain was 6.45 ± 1.29 before surgery, 1.63 ± 0.81 at the second day, 1.55 ± 0.82 at 3 months, 1.45 ± 0.93 at 6 months and 1.36 ± 1.29 at 12 months after surgery. The ODI values were 60.09 ± 5.61 before surgery, 21.36 ± 6.99 on second day, 20.91 ± 7.03 at 3 month, 20.27 ± 7.46 at 6 months and 18.64 ± 6.50 at 12 months after surgery. The mean preoperative JOA score was 12.55 ± 1.57, which increased to 16.09 ± 1.04 on second day, 16.18 ± 1.40 at 3 months, 16.27 ± 1.74 at 6 months and 17.91 ± 1.92at 12 months after surgery. The differences in the scores before and after surgery were statistically significant (*P* < 0.001, Table 2, Fig. 7). All 11 patients showed satisfactory effects according to the modified MacNab criteria by the last follow-up visit (Table 3).

**Table 2:** Comparison of pre- and postoperative VAS, JOA and ODI scores

Timepoint	VAS leg pain scores	VAS back pain scores	JOA scores	ODI (%)
Pre-operation	6.18 ± 1.54	6.45 ± 1.29	12.55 ± 1.57	60.09 ± 5.61
Post-operation				
2 d	2.09 ± 1.14	1.63 ± 0.81	16.09 ± 1.04	21.36 ± 6.99
3 m	1.91 ± 0.94	1.55 ± 0.82	16.18 ± 1.40	20.91 ± 7.03
6 m	1.82 ± 1.08	1.45 ± 0.93	16.27 ± 1.74	20.27 ± 7.46
12 m	1.73 ± 1.10	1.36 ± 1.29	17.91 ± 1.92	18.64 ± 6.50
*F*	101.746	146.988	25.996	357.215
*P*	P < 0.001	P < 0.001	P < 0.001	P < 0.001

**Table 3:** Macnab outcome evaluated at the final visit of the follow-up

Total	Excellent	Good	Fair	Poor
11	10	1	0	0

Excellent: free of pain and deficit, without restriction of mobility;

Good: residual symptoms or deficits not impeding a normal life;

Fair: some improvement in functionality but remained handicapped;

Poor: no improvement at all

### Perioperative complications

There were no complications, such as nerve injury, CSF leakage or wound infection in our series. Three cases had transient paresthesias after surgery which disappeared in 2 to 3 weeks. There were no cases of instability or further recurrence at the time of final follow-up.

### Representative cases

#### Representative cases are presented in Fig. 8 and Fig. 9

**Fig. 8:**
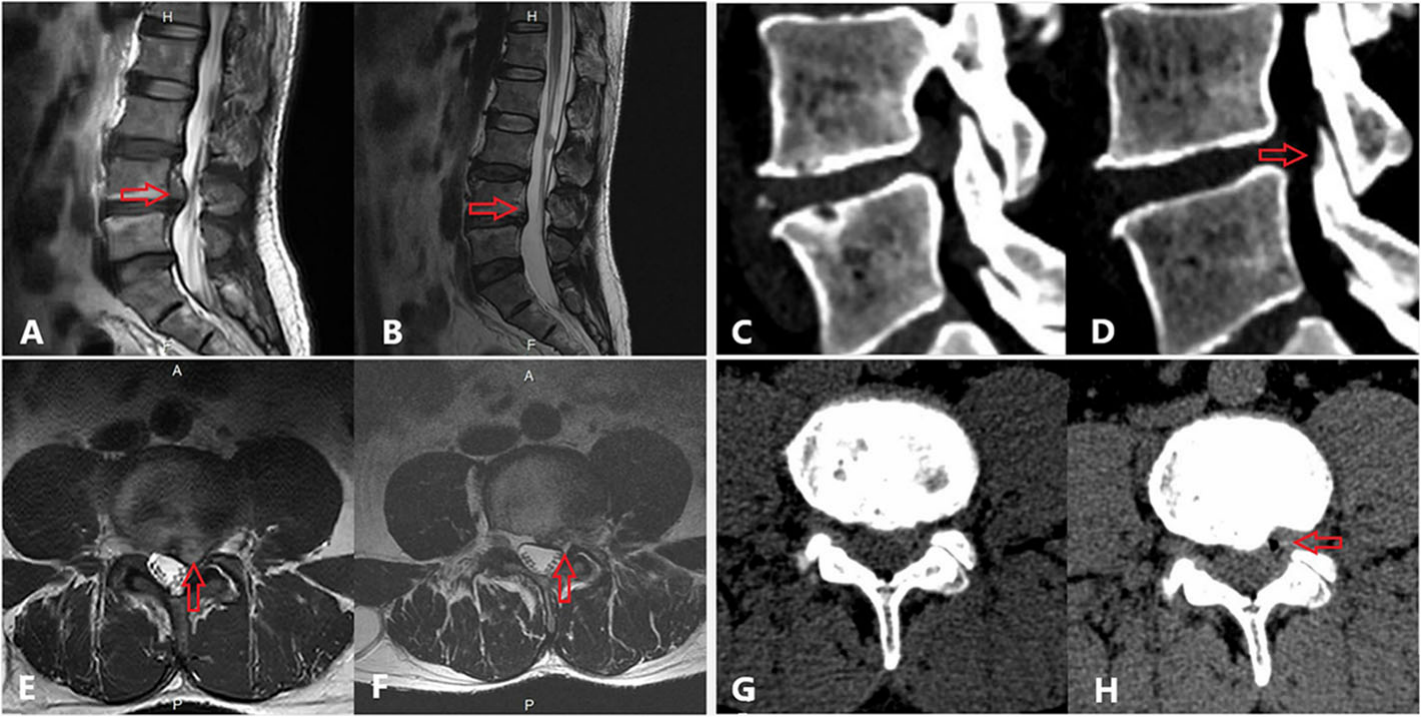
The preoperative and postoperative imaging data of patient who received VTT involving TELF. (**A**) preoperative sagittal MRI image showed L4-L5 very highly up-migrated lumbar disc herniation. (**B**) postoperative sagittal MRI image showed that the protruding tissue in the spinal canal was completely removed. (**C**) preoperative sagittal CT reconstruction. (**D**) postoperative sagittal CT reconstruction demonstrated the ventral part of SAP of the inferior vertebra be partially resected, not offending the articular surface. (**E**) preoperative axial MRI image showed L4-L5 very highly up-migrated lumbar disc herniation located in left side. (**F**) postoperative axial MRI image showed that the protruding tissue in the spinal canal was completely removed. (**G**) preoperative axial CT at L4 level. (**H**) postoperative axial CT demonstrated the trench which we dig in the posterior surface of L4 vertebral body

A 58-year-old man suffered from low back pain, left gluteal, thigh and calf pain for 2 months. The manual muscle test for the left great-toe dorsiflexion showed grades IV. His symptoms had worsened despite conservative treatment. On preoperative physical examination, the femoral nerve stretch test was positive, and the left straight leg raise test was restricted to within 20°. The magnetic resonance (MR) images demonstrated the disc extrusion and the distant upward migrated disc herniation at the L4–5 level (Fig. 8A, E). The patient underwent VTT involving TELF to remove the L4/5 distant upward migrated herniated disc. MRI was immediately repeated, which showed that the protruding tissue in the spinal canal was completely removed (Fig. 8B, F). Preoperative (Fig.8C,G) and Postoperative CT scan (Fig. 8D,H)showed the bone resection of TELE and VTT.

**Fig. 9:**
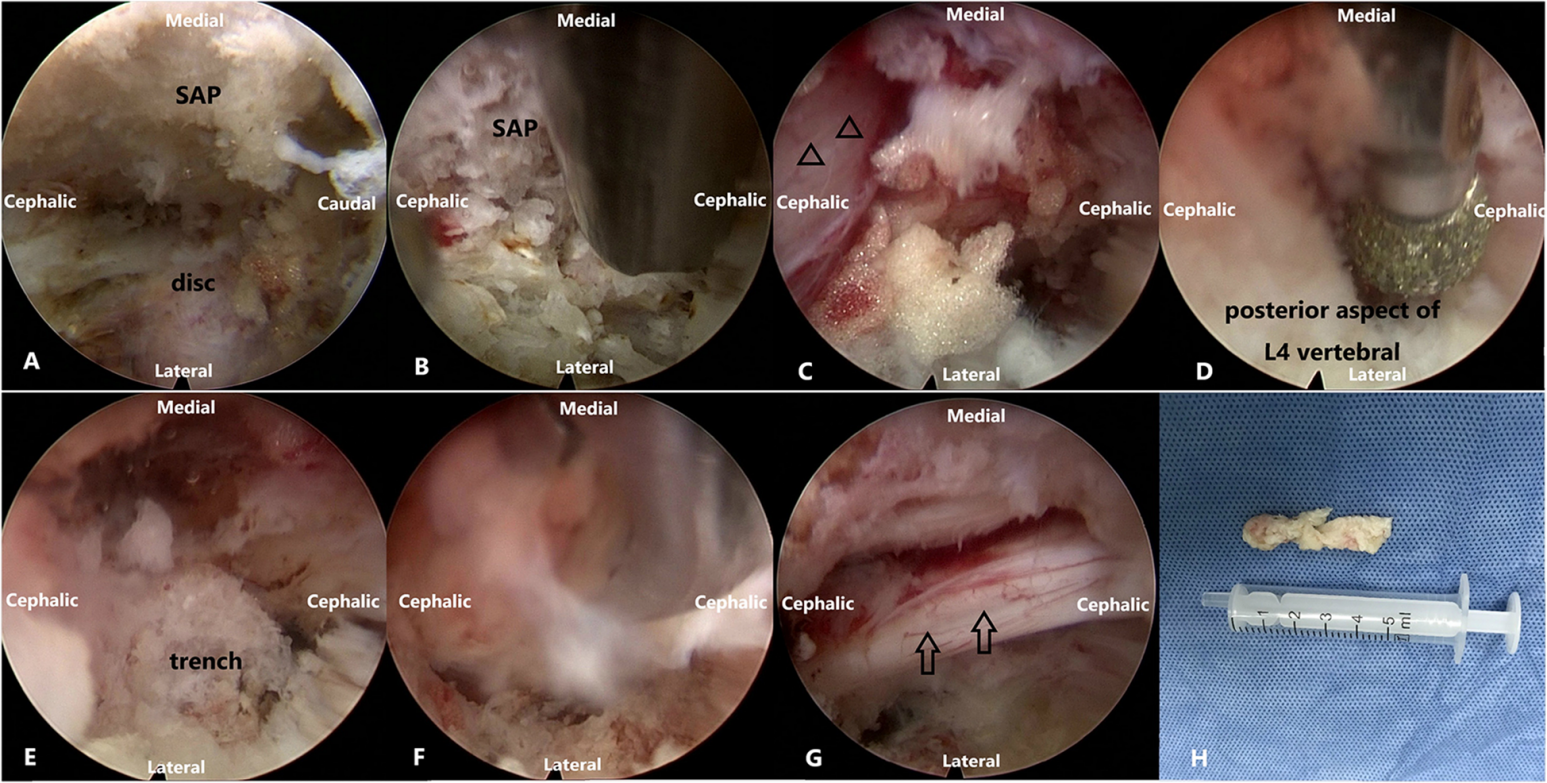
Intraoperative endoscopic view. (**A**) The foramina zone was exposed before the full-endoscopic visualized foraminotomy was performed. (SAP = Superior articular process); (**B**) Full-endoscopic visualized foraminotomy performed with ultrasonic bone knife; (**C**) the exiting nerve root(triangle)was exposed; (**D**) and E. The diamond bur was used to dig a trench at the posterior aspect of L4 vertebral; (**F**) The endoscope with working cannula moved into the trench. The herniated nucleus pulposus was extracted; (**G**) Sufficient decompression of the traversing nerve root(arrow)was ensured; (**H**) The specimen

## Discussion

The philosophy and strategy of the treatment to LDH have undergone tremendous changes in recent years [2, 3, 31]. The PELD has gradually become well accepted for providing several theoretical advantages over conventional open surgery [12, 14–17]. With the great improvement of instrumentations including high-definite endoscopes, radiofrequency probes, bone drills, ultrasonic bone knife and articulated flexible forceps, as well as a better understanding of endoscopic surgical approaches, PELD has gradually become the preferred choice for the treatment of LDH, and a variety of surgical approaches are currently available [2, 3].

However, LDHs with high-grade migrated (upward or downward) are still considered to be challenging under PELD even for experienced surgeon. Choi et al. analyzed 10,228 patients with LDH who underwent PTELD. Among the 283 postoperative residual disc tissue cases, 70 were identified as migrated herniation (24.7%), and 11 cases were identified as distant migrated herniation (3.9%) [20]. Indeed, disc migration is a very frequent event, and downward migrated fragments are more common than upward ones [32, 33]. Upward herniations are commonly sequestrated which can migrate up to midlevel of vertebra and may be separated into multiple fragments [33]. For this reason, those fragmented herniations could not be completely removed just by grasping the proximal part of the herniation [27]. Herniations with very highly up-migrated tend to lie under the pars interarticularis medial to the pedicle, which is farther away from working cannula and endoscopic apparatus. What’s more difficult is the obstacle of the exiting nerve. When rotating and shifting the working cannula upward, the exiting nerve may be irritated and injured. Therefore, all of above factors contribute to the possibility of incomplete removal the very highly up-migrated mass. Lee et al. concluded that LDH with downward migration could be treated with traditional PELD, but for highly up-migrated, PELD was associated with a high risk of postoperative residual herniated disc tissue. Thus, open surgery was a more secure option [21] .

When these fragments approached by posterior open discectomy, especially the sequestrated ones, it is inevitable to retract paravertebral muscle, remove the bone in the vital region of pars or facets which may destabilize the motion segment to aggravate chronic back pain [34]. Up-migrated herniations and sequestrations are also more common in elderly patients with associated medical comorbidities, which make them unsuitable for general anesthesia and open surgery [35, 36]. In our series, the patients’ average age was 68.5 and all the patients could tolerate the surgery under local anesthesia and fell well during the operation.

PELD can be performed under local anesthesia and is definitely advantageous in these cases. Transforaminal PELD is the most common approach on the basis of the Kambin’s triangle. In order to improve the efficacy of the procedure to get promising surgical results, we ameliorated the endoscopic techniques, which comprises two key steps: 1) the full-endoscopic visualized foraminotomy was achieved with the help of burr or ultrasonic bone knife. We performed the full-endoscopic visualized foraminotomy with the aid of excellent endoscopic visualization rather than with the aid of intervention technique. Hence, the fluoroscopy time and radiation exposure can be reduced significantly. Endoscopic visualization can also help us to control the boundary of bone resection according to our needs, avoid removing too much bone and damaging the articular surface [21, 29]. Furthermore, the trajectory of the full-endoscopic visualized foraminotomy is cranio-caudal approach angle, which is familiar to surgeons, can keep the puncture needle, pencil-like rod and working cannula away from the exiting nerve without irritation. After foraminotomy, enough manipulation space is obtained. The endoscope can be advanced into the spinal canal and gradually turned to the cranial direction. 2) drilling a trench in the posterior surface of the superior vertebral body. With the help of the trench, the working cannula with endoscopy can crawl upward further and get closer to the target without offending the nerves and thecal sac. The angled forceps were used to clamp the nucleus pulposus and remove it in the trench. The procedure was under the direct vision to ensure no residue. With the application of the trench technique, a sufficient path and space can be obtained to reach the level of the inferior margin of superior pedicle. The establishment of an ideal minimally invasive spinal surgical pathway means that it should be directly aimed at the surgical target as close as possible. Our surgical method meets this requirement. In our surgical cases, postoperative MRI demonstrated complete decompression of the HNP and no missing fragment. The overall success rate was 100%. According to modified MacNab criteria, the final follow-up was excellent in 10 of 11 patients (90.9%) and good in1 patient (9.1%), and there was none in poor.

There were some other approaches have been reported. Kim et al. removed very highly up-migrated discs through the contralateral foramen approach. However, this method required a longer working channel and was associated with a possible increased risk of injury [28]. Transpedicular approach have been used successfully to remove the highly migrated discs. However, the herniated fragments migrated caudally in most of the reported cases [37]. Theoretically, the disc fragments on the shoulder aspect of traversing root may be the first-rate indication because it is in close proximity to the medial wall of pedicle. In contrast, some authors reported that the axillary highly up-migrated disc herniations also can be removed successfully [22]. The relative contraindications are hypoplastic pedicle, severe canal stenosis and calcified disc fragments, etc. The posterior approaches are good alternative including -interlaminar approach [19, 27] and modified translaminar Osseous Channel approaches [25, 26]. For L5-S1 highly upward migrated herniation, we routinely used the posterior interlaminar approach which enable to avoid the obstruction of iliac crest and the embarrassment of working channel angle. The interlaminar window is large and can be further enlarged cranially with endoscopic drill/Kerrison punch, through which the migrated disc material can be removed successfully [27]. For L4–5 and above(L3–4\L2–3), the level of interlaminar space is below the level of the disc apace, increasing the difficulty of gaining access to the very highly upward migrated herniation via an interlaminar approach. A narrow interlaminar space may also restrict the manipulation of surgical instruments. In addition, to get access to the highly upward migrated fragment, an extensive laminectomy may be necessary to approach the distal part of the disc fragments, which is more time-consuming, can cause bleeding. Establish a bone tunnel in the laminar is a translaminar approach. But it is not a routine approach. Specific care must be taken because this area between the lamina and dura mater does not contain ligament flavum. As a result, careless maneuvering may cause a dura tear or nerve root injury [38]. Furthermore, the interlaminar approaches usually necessitate general anesthesia. In our case series, the patients could tolerate the surgery under local anesthesia and fell well during the operation. It benefits for rapid recovery.

Recently, the isthmus foraminotomy technique have been reported for the successful treatment of very highly up-migrated lumbar disc herniation, which the target point is isthmus instead of ventral portion of superior articular process [38]. Since the lateral isthmus is resected and the upper intervertebral foramen is enlarged, the space between the dura sac and exiting nerve root is under endoscopic visualization. This technique can obtain initial cranial approach angle. However, the isthmus foraminotomy is not like classic foraminotomy, which is familiar to most of the spine surgeon. The target of isthmus foraminotomy is cranial half of the foramen, relatively compacted with neurovascular structures. So, theoretically, this technique has more risk to injury the exiting root during reaming procedure and inserting the working cannula. According to the literature, the protective sleeve which is special designed should be used.

Of course, our investigation has certain limitations: [1] this was a retrospective study of small sample size. Additional samples are necessary before widespread application is possible [2]. The short follow-up limited our ability to observe the long-term efficacy of the procedure and complications [3]. This technique can only be used as a supplement to the conventional surgical procedure for the very highly upward migrated herniation. Furthermore, the application of this procedure must be based on careful preoperative planning and extensive experience in PELD.

## Conclusion

The novel VTT technique involving TELF is a supplement to the traditional PELD. This technique enables the endoscope with working cannula to get closer to the SNPs, and the latter can be removed successfully under direct visualization. After a surgeon becomes proficient in PELD via the conventional approach, the VTT technique involving TELF can be a safe, effective and feasible surgical procedure for the treatment of LDH with very highly upward migrated.

## Data Availability

The datasets used or analyzed during the current study are available from the corresponding author on reasonable request.

## Abbreviations

VTT: vertebral trench technique
TELF: transforaminal endoscopic lumbar foraminotomy
VHUM-LDH: very highly up-migrated lumbar disc herniation
PELD: percutaneous endoscopic lumbar discectomy
LDH: lumbar disc herniation
VAS: visual analogue score
ODI: Oswestry Disability Index
JOA: Japanese Orthopaedic Association
SNPs: sequestrated nucleus pulposus
SAP: superior articular process

## References

[1] Yoon JW, Wang MY (2019). The evolution of minimally invasive spine surgery. J Neurosurg Spine.

[2] Hasan S, Hartl R, Hofstetter CP (2019). The benefit zone of full-endoscopic spine surgery. J Spine Surg.

[3] Choi G, Pophale CS, Patel B, Uniyal P (2017). Endoscopic spine surgery. J Korean Neurosurg Soc.

[4] Mayer HM (2019). A history of endoscopic lumbar spine surgery: what have we learnt?. Biomed Res Int.

[5] Schreiber A, Suezawa Y, Leu H (1989). Does percutaneous nucleotomy with discoscopy replace conventional discectomy? Eight years of experience and results in treatment of herniated lumbar disc. Clin Orthop Relat Res.

[6] Kambin P, Gellman H. Percutaneous lateral discectomy of the lumbar spine a preliminary report. Clin Orthop Relat Res. 1983;174.

[7] Schaffer JL, Kambin P (1991). Percutaneous posterolateral lumbar discectomy and decompression with a 6.9-millimeter cannula. Analysis of operative failures and complications. J Bone Joint Surg Am.

[8] Yeung AT, Tsou PM (2002). Posterolateral endoscopic excision for lumbar disc herniation: surgical technique, outcome, and complications in 307 consecutive cases. Spine (Phila Pa 1976).

[9] Kim M, Kim HS, Oh SW, Adsul NM, Singh R, Kashlan ON, Noh JH, Jang IT, Oh SH (2019). Evolution of spinal endoscopic surgery. Neurospine..

[10] Kim HS, Paudel B, Jang JS, Lee K, Oh SH, Jang IT (2018). Percutaneous endoscopic lumbar discectomy for all types of lumbar disc Herniations (LDH) including severely difficult and extremely difficult LDH cases. Pain Physician.

[11] Fiorenza V, Ascanio F (2019). Percutaneous endoscopic Transforaminal outside-in outside technique for Foraminal and Extraforaminal lumbar disc Herniations-operative technique. World Neurosurg.

[12] Liu X, Yuan S, Tian Y, Wang L, Gong L, Zheng Y, Li J (2018). Comparison of percutaneous endoscopic transforaminal discectomy, microendoscopic discectomy, and microdiscectomy for symptomatic lumbar disc herniation: minimum 2-year follow-up results. J Neurosurg Spine..

[13] Lin GX, Kotheeranurak V, Mahatthanatrakul A, Ruetten S, Yeung A, Lee SH, Ahn Y, Kim HS, Hofstetter C, Lee JH, Choi KC, Lewandrowski KU, Kim JS (2020). Worldwide research productivity in the field of full-endoscopic spine surgery: a bibliometric study. Eur Spine J.

[14] Ruetten S, Komp M, Merk H, Godolias G (2009). Surgical treatment for lumbar lateral recess stenosis with the full-endoscopic interlaminar approach versus conventional microsurgical technique: a prospective, randomized, controlled study. J Neurosurg Spine..

[15] Komp M, Hahn P, Oezdemir S, Giannakopoulos A, Heikenfeld R, Kasch R, Merk H, Godolias G, Ruetten S (2015). Bilateral spinal decompression of lumbar central stenosis with the full-endoscopic interlaminar versus microsurgical laminotomy technique: a prospective, randomized, controlled study. Pain Physician..

[16] Ruetten S, Komp M, Merk H, Godolias G (2008). Full-endoscopic interlaminar and transforaminal lumbar discectomy versus conventional microsurgical technique: a prospective, randomized, controlled study. Spine (Phila Pa 1976).

[17] Kim M, Lee S, Kim HS, Park S, Shim SY, Lim DJ (2018). A comparison of percutaneous endoscopic lumbar discectomy and open lumbar microdiscectomy for lumbar disc herniation in the Korean: a Meta-analysis. Biomed Res Int.

[18] Ruetten S, Komp M, Merk H, Godolias G (2007). Use of newly developed instruments and endoscopes: full-endoscopic resection of lumbar disc herniations via the interlaminar and lateral transforaminal approach. J Neurosurg Spine..

[19] Choi G, Lee SH, Raiturker PP, Lee S, Chae YS. Percutaneous endoscopic interlaminar discectomy for intracanalicular disc herniations at L5-S1 using a rigid working channel endoscope. Neurosurgery*.* 2006;58(1 Suppl):ONS59–68; discussion ONS59–68. 10.1227/01.neu.0000192713.95921.4a 16479630

[20] Choi KC, Lee JH, Kim JS, Sabal LA, Lee S, Kim H, et al. Unsuccessful percutaneous endoscopic lumbar discectomy: a single-center experience of 10,228 cases. Neurosurgery. 2015;76(4):372–80; discussion 380-371; quiz 381. 10.1227/NEU.0000000000000628. 10.1227/NEU.0000000000000628 25599214

[21] Lee S, Kim SK, Lee SH, Kim WJ, Choi WC, Choi G, Shin SW (2007). Percutaneous endoscopic lumbar discectomy for migrated disc herniation: classification of disc migration and surgical approaches. Eur Spine J.

[22] Krzok G, Telfeian AE, Wagner R, Iprenburg M (2016). Transpedicular lumbar endoscopic surgery for highly migrated disk extrusions: preliminary series and surgical technique. World Neurosurg..

[23] Quillo-Olvera J, Akbary K, Kim JS (2018). Percutaneous endoscopic transpedicular approach for high-grade down-migrated lumbar disc herniations. Acta Neurochir.

[24] Hu QF, Pan H, Fang YY, Jia GY (2018). Percutaneous endoscopic lumbar discectomy for high-grade down-migrated disc using a trans-facet process and pedicle-complex approach: a technical case series. Eur Spine J.

[25] Xin Z, Liao W, Ao J (2017). A modified translaminar Osseous Channel-assisted percutaneous endoscopic lumbar discectomy for highly migrated and sequestrated disc Herniations of the upper lumbar: clinical outcomes, surgical indications, and technical considerations. Biomed Res Int.

[26] Xia Y, Zhang Q, Gao X, Wang K, Zhang X, du Y, Chen L (2019). Posterior percutaneous endoscopic lumbar discectomy combined with the vertical anchoring technique for lumbar disc herniation with distant upward migration. J Orthop Surg Res.

[27] Kim CH, Chung CK, Woo JW (2016). Surgical outcome of percutaneous endoscopic Interlaminar lumbar discectomy for highly migrated disk herniation. Clin Spine Surg.

[28] Kim JS, Choi G, Lee SH (2011). Percutaneous endoscopic lumbar discectomy via contralateral approach: a technical case report. Spine (Phila Pa 1976).

[29] Hua W, Zhang Y, Wu X, Gao Y, Li S, Wang K, Yang S, Yang C (2019). Full-endoscopic visualized Foraminoplasty and discectomy under general anesthesia in the treatment of L4-L5 and L5-S1 disc herniation. Spine (Phila Pa 1976).

[30] Morishita Y, Hida S, Naito M, Arimizu J, Matsushima U, Nakamura A (2006). Measurement of the local pressure of the intervertebral foramen and the electrophysiologic values of the spinal nerve roots in the vertebral foramen. Spine (Phila Pa 1976).

[31] Kreiner DS, Hwang SW, Easa JE (2014). An evidence-based clinical guideline for the diagnosis and treatment of lumbar disc herniation with radiculopathy. Spine J.

[32] Kuzeyli K, Cakir E, Usul H (2003). Posterior epidural migration of lumbar disc fragments: report of three cases. Spine (Phila Pa 1976).

[33] Schellinger D, Manz HJ, Vidic B, Patronas NJ, Deveikis JP, Muraki AS, Abdullah DC (1990). Disk fragment migration. Radiology..

[34] Osman SG, Nibu K, Panjabi MM, Marsolais EB, Chaudhary R. Transforaminal and posterior decompressions of the lumbar spine. A comparative study of stability and intervertebral foramen area. Spine (Phila Pa 1976)*.* 1997;22(15):1690–95. 10.1097/00007632-199708010-00002 9259777

[35] An HS, Vaccaro A, Simeone FA, Balderston RA, O'Neill D (1990). Herniated lumbar disc in patients over the age of fifty. J Spinal Disord.

[36] Akagi S, Saito T, Kato I, Sasai K, Ogawa R (2000). Clinical and pathologic characteristics of lumbar disk herniation in the elderly. Orthopedics..

[37] Giordan E, Del Verme J, Coluzzi F (2020). Full-endoscopic transpedicular discectomy (FETD) for lumbar herniations: case report and review of the literature. Int J Surg Case Rep.

[38] Li Z-Z, Ma S-Y, Cao Z, Zhao HL (2020). Percutaneous isthmus Foraminoplasty and full-endoscopic lumbar discectomy for very highly Upmigrated lumbar disc herniation: technique notes and 2 years follow-up. World Neurosurgery.

